# Peptide‐based therapeutic cancer vaccine: Current trends in clinical application

**DOI:** 10.1111/cpr.13025

**Published:** 2021-03-22

**Authors:** Wensi Liu, Haichao Tang, Luanfeng Li, Xiangyi Wang, Zhaojin Yu, Jianping Li

**Affiliations:** ^1^ Department of Pharmacology School of Pharmacy China Medical University Shenyang China; ^2^ Liaoning Key Laboratory of molecular targeted anti‐tumor drug development and evaluation Liaoning Cancer immune peptide drug Engineering Technology Research Center Shenyang China; ^3^ Transfusion Medicine Institute Liaoning Blood Center Shenyang China; ^4^ Transfusion Medicine Institute Harbin Blood Center Harbin China

**Keywords:** adjuvant and nanomaterial, cancer immunotherapy, combination strategy, epitope peptides, peptide‐based therapeutic cancer vaccine

## Abstract

The peptide‐based therapeutic cancer vaccines have attracted enormous attention in recent years as one of the effective treatments of tumour immunotherapy. Most of peptide‐based vaccines are based on epitope peptides stimulating CD8^+^ T cells or CD4^+^ T helper cells to target tumour‐associated antigens (TAAs) or tumour‐specific antigens (TSAs). Some adjuvants and nanomaterials have been exploited to optimize the efficiency of immune response of the epitope peptide to improve its clinical application. At present, numerous peptide‐based therapeutic cancer vaccines have been developed and achieved significant clinical benefits. Similarly, the combination of peptide‐based vaccines and other therapies has demonstrated a superior efficacy in improving anti‐cancer activity. We delve deeper into the choices of targets, design and screening of epitope peptides, clinical efficacy and adverse events of peptide‐based vaccines, and strategies combination of peptide‐based therapeutic cancer vaccines and other therapies. The review will provide a detailed overview and basis for future clinical application of peptide‐based therapeutic cancer vaccines.

## Graphical abstract

Despite few clinical successes, peptide‐based therapeutic cancer vaccines have raised hopes in recent years. Therefore, better understanding of immune response, clinical benefits and adverse events, the formulations, and the promising combination strategies with conventional therapies foster development of the next generation of peptide‐based cancer vaccines.

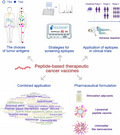

**Abbreviations**
AE: adverse event
ANN: artificial neural network
CEA: carcinoembryonic antigen
CpG ODN: cytosine guanine oligodeoxynucleotide
CR: complete response
CTL: cytotoxic T lymphocyte
DC: dendritic cell
DSPC: distearoyl phosphatidylcholine
DSPG: distearoyl phosphatidylglycerole
FDA: Food and Drug Administration
GM‐CSF: granulocyte‐macrophage colony‐stimulating factor
HLA: human leucocyte antigen
HLA‐I: HLA class I antigen
HLA‐II: HLA class II antigen
IDO: indoleamine 2,3‐dioxygenase
IEDB: the immune epitope database
IFA: incomplete Freund adjuvant
IFN‐γ: interferon‐γ
IL‐2: interleukin‐2
ISA: incomplete Seppic adjuvant
LPS: lipopolysaccharides
MAGE‐1: melanoma antigen‐1
MAGE‐A1: melanoma antigen‐A1
MART‐1: melanoma antigen recognized by T cells 1
MHC‐I: major histocompatibility complex I
MHC‐II: major histocompatibility complex II
MPLA: monophosphoryl lipid A
MUC: mucin
OS: overall survival
PD‐1: programmed death 1
**poly‐ICLC**: lysine and carboxymethylcellulose
ppCT: preprocalcitonin
PPV: personalized peptide vaccination
PR3: proteinase‐3
PSSM: position‐specific scoring matrix
RAI: reaction at the injection sites
RFS: disease‐free survival
RT: radiotherapy
TA: tumour antigen
TAA: tumour‐associated antigen
TAP: transporter associated with antigen processing
TEIPP: T‐cell epitopes associated with impaired peptide processing
TIL: tumour infiltrating lymphocyte
TLR: toll‐like receptor
TSA: tumour‐specific antigen
TTK: TTK protein kinase
VEGFR: vascular endothelial growth factor receptor

## 1 INTRODUCTION

Immunotherapeutic strategies have dramatically revolutionized cancer treatments, including dendritic cell (DC)‐based cancer vaccines, immune checkpoint inhibitors and chimeric antigen receptor T‐cell immunotherapies (CAR‐T). For example, checkpoint inhibitor‐based immunotherapies that could activate T cells result in an improvement in clinical success, but the tumour targeting was deficient. Despite specific tumour targeting, CAR‐T therapy showed risks of cytokine release syndrome and neurotoxicity, and it could not gain clinical benefits on solid tumours, which caused the limitation of clinical application. Therefore, developing the safe and effective treatments to enhance the specific anti‐tumour activity has become a hot topic in the current field of tumour immunotherapy.

The peptide‐based therapeutic cancer vaccines could offer many advantages with regard to convenient production, cost‐effective manufacture, low carcinogenic potential, insusceptible pathogen contamination and high chemical stability. This type of vaccine contains the distinct 8‐12 aa peptide from tumour antigen (TA) coding sequence. TAs are formed by overexpressing and emerging proteins during the process of tumorigenesis and development. It could be internalized into DCs, where they are degraded into peptides and assemble to human leucocyte antigen (HLA) molecules on DCs surface for T‐cell activation. HLA is the expression product of the human major histocompatibility complex (MHC), which is related to immune response. Regarding the interaction of T cells and DCs, T cells not only recognize specific TA but also recognize the distinct peptide‐HLA complex. The strategy of identifying novel peptides from TA is an attractive method for immunotherapy with clinical benefit and cost‐effectiveness.

Furthermore, the mode of administration is easy and the immune response could be monitored in vitro; thereby, peptide‐based therapeutic cancer vaccines could be a promising approach for cancer therapies. These parameters to develop peptide‐based therapeutic cancer vaccines are critical, such as choices of proper tumour antigens, effective screening and modification methods of epitope peptides, and selections of proper formulations. Furthermore, growing evidence has demonstrated that combination between peptide‐based vaccines and other therapies could offer an ideal view of cancer immunotherapy. In the review, we discussed multiple peptide‐based therapeutic cancer vaccines in various cancer types and their immune response and clinical benefits.

## 2 TARGET CHOICES OF PEPTIDE‐BASED THERAPEUTIC CANCER VACCINES

The CD8^+^ T cells are capable of recognizing the peptide‐HLA complex to produce a persistent memory CTL response against target cells expressing the antigen. Therefore, the critical factor is the selection of proper TA for therapeutic cancer vaccines to exert specific cytotoxicity against tumour cells.

**FIGURE 1:**
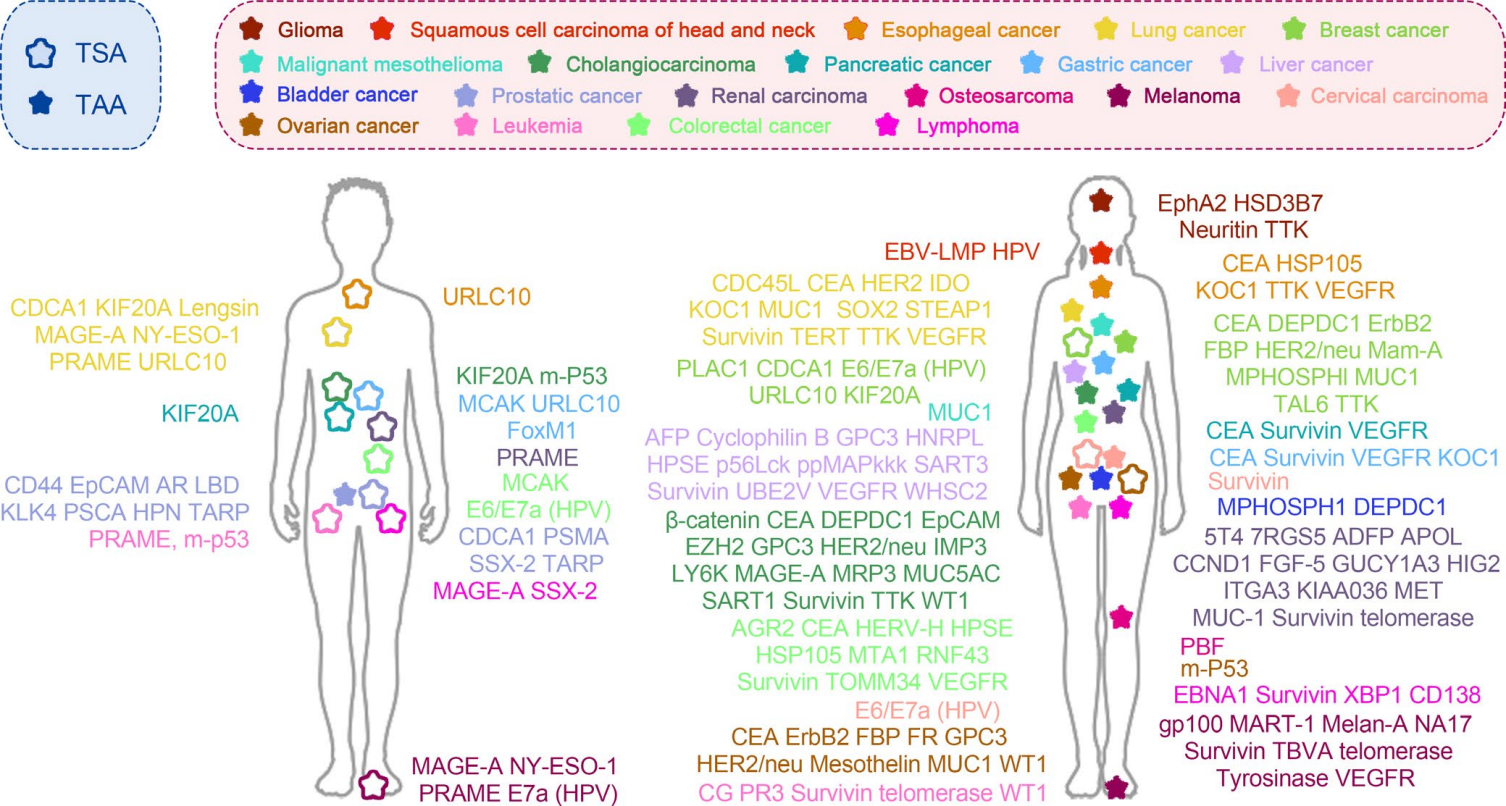
Tumour antigens for peptide‐based therapeutic cancer vaccine in different types of tumours

**FIGURE 2:**
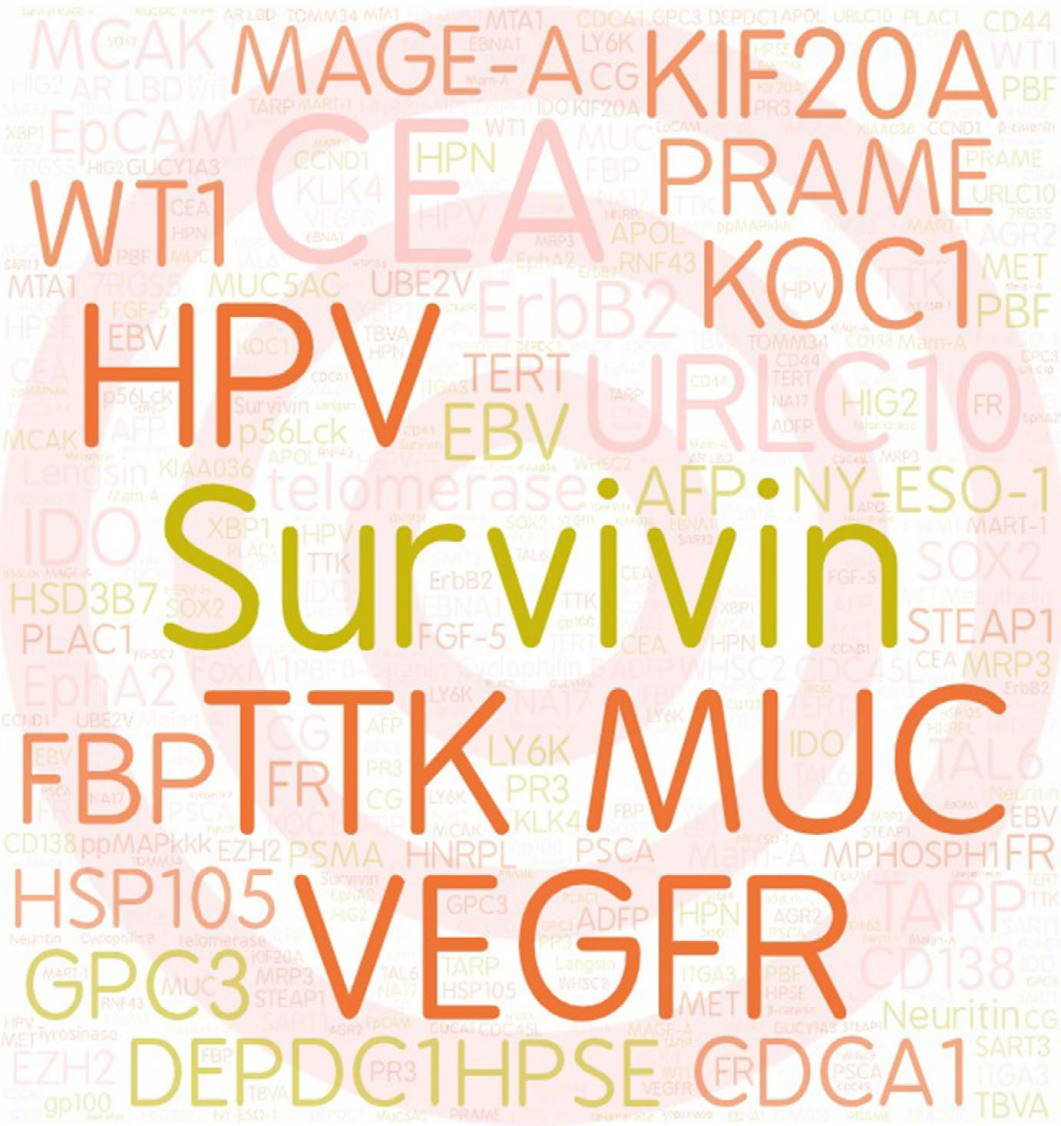
Frequency of tumour antigens using in clinical trials for peptide‐based therapeutic cancer vaccines

TAs can be classified into tumour‐associated antigens (TAA) and tumour‐specific antigens (TSA). Despite TAAs can express in both normal cells and tumour cells, they overexpress in tumour cells but at a low level in normal cells. Therefore, TAAs are attractive targets for developing immunotherapeutic cancer vaccines. Some studies reported that characteristics of proper TAA should be the following: i) differential expression between normal cells and tumour cells; ii) involvement in cell cycle; and iii) association with cell survival.^1^ Normally, most of TAAs with low self‐tolerance and strong immunogenicity were used as targets in preclinical studies and clinical trials to evaluate safety and efficacy of peptide‐based therapeutic cancer vaccines.^2^ On the other hand, TSA only expressed in tumour cells rather than in normal cells, including mutations of normal proteins,^3^ cancer testis antigen,^4^, ^5^ neoantigens^6^ and virus‐related antigens.^7^, ^8^ Boon et al reported melanoma antigen‐A1 (MAGE‐A1) as the first TSA in humans at 1991.^9^ Human leucocyte antigen (HLA) / TSA‐derived peptide complex, could exert higher avidity specific T cells to lead to effective and safe immune response of cancer vaccines against tumour.^10^, ^11^ TSAs as targets of cancer vaccines demonstrated similar results in both animal models and clinical trials due to the loss of TSA expression in normal tissues, which means non‐immunologically tolerant to TSA and non‐immunity targeting normal tissues.^12^ TSAs are attractive for personalized cancer immunotherapy, but it is not cost‐effective.^13^ Besides, some studies emerge for the selection of specific epitopes, such as T‐cell epitopes associated with impaired peptide processing (TEIPP),^14^ which only express on transporter associated with antigen processing (TAP)‐deficient tumour cell surface. The preprocalcitonin (ppCT) _16–25_ antigenic peptide, derived from the calcitonin hormone precursor, as the first human TEIPP Ag, provides a new strategy to counteract immune evasion by antigenic processing machinery defects.^15^ Currently, many TAAs and TSAs have been identified as targets for peptide‐based therapeutic cancer vaccines (Figure 1, Table 1), in which most focus on targeting melanoma,^16^ lung cancer,^17^ breast cancer^18^ and leukaemia,^19^, ^20^ whereas most of them are in phase I and phase II. Recent clinical trials in phase III are only including HER2 (human epidermal growth factor receptor 2)/neu targeting breast cancer^21^; tyrosinase, gp100 antigen, and MART‐1 (melanoma antigen recognized by T cells 1) antigen targeting melanoma; PR3 (proteinase‐3) targeting leukaemia.^22^ TAs, such as Survvin, VEGFR (vascular endothelial growth factor receptor), MUC1 (mucin 1) and TTK (TTK protein kinase), were used most extensively as targets for developing peptide‐based therapeutic cancer vaccines, targeting lung cancer, gastrointestinal cancer and melanoma (Figure 2).

**TABLE 1:** Antigens for peptide‐based therapeutic cancer vaccines

Tumour	Tumour‐specific antigens	Tumour‐associated antigens
Glioma		EphA2, HSD3B7, Neuritin, TTK
Squamous cell carcinoma of head and neck		EBV‐LMP, HPV
Oesophageal cancer	URLC10	CEA, HSP105, KOC1,TTK, VEGFR
Lung cancer	CDCA1, KIF20A, Lengsin, MAGE‐A, NY‐ESO‐1, PRAME, URLC10	CDC45L, CEA, HER2, IDO, KOC1, MUC1, SOX2, STEAP1, Survivin, TERT, TTK, VEGFR
Breast cancer	PLAC1, CDCA1, E6/E7a (HPV), URLC10, KIF20A, m‐P53	CEA, DEPDC1, ErbB2, FBP, HER2/neu, Mam‐A, MPHOSPHl, MUC1, TAL6,TTK
Malignant pleural mesothelioma		MUC1
Liver cancer		AFP, Cyclophilin B, GPC3, HNRPL, HPSE, p56Lck, ppMAPkkk, SART3, Survivin, UBE2V, VEGFR, WHSC2
Cholangiocarcinoma	KIF20A, MAGE‐A, m‐P53	β‐catenin, CEA, DEPDC1, EpCAM, EZH2, GPC3, HER2/neu, IMP3, LY6K, MRP3, MUC5AC, SART1, Survivin, TTK, WT1
Pancreatic cancer	KIF20A	CEA, Survivin, VEGFR
Gastric cancer	MCAK, URLC10, FoxM1	CEA, Survivin, VEGFR, KOC1
Bladder cancer		MPHOSPH1, DEPDC1
Prostatic cancer	CDCA1, PSMA, SSX‐2, TARP	CD44, EpCAM, AR LBD, KLK4, PSCA, HPN
Renal carcinoma	PRAME	5T4, 7RGS5, ADFP, APOL, CCND1, FGF‐5, GUCY1A3, HIG2, ITGA3, KIAA036, MET, MUC1, Survivin, telomerase
Osteosarcoma		PBF
Melanoma	MAGE‐A, NY‐ESO‐1, PRAME, E7a (HPV)	gp100, MART‐1, Melan‐A, NA17, Survivin, TBVA, telomerase, Tyrosinase, VEGFR
Cervical carcinoma	E6/E7a (HPV)	Survivin
Ovarian cancer	m‐P53	CEA, ErbB2, FBP, FR, GPC3, HER2/neu, Mesothelin, MUC1, WT1
Leukaemia	PRAME, m‐p53	CG, PR3, Survivin, telomerase, WT1
Colorectal cancer	MCAK, E6/E7a (HPV)	AGR2, CEA, HERV‐H, HPSE, HSP105, MTA1, RNF43, Survivin, TOMM34, VEGFR
Lymphoma	MAGE‐A, SSX‐2	EBNA1, Survivin, XBP1, CD138

## 3 STRATEGIES FOR SCREENING EPITOPE PEPTIDES

The anti‐tumour effects of DC‐mediated T‐cell activation are through the stimulation of peptides, terms epitopes, instead of the entire antigen molecule. Normally, the epitope for developing peptide‐based therapeutic cancer vaccines is a short amino sequence derived from TA with immunogenicity and HLA allele compatibility. It has been reported many screening strategies for immunodominant epitopes, such as bioinformatic analysis and HLA ligandome. The affinity of HLA‐I allele and epitopes can be measured and predicted by many methods (Table 2), including the method based on structural analysis, the position‐specific scoring matrix (PSSM), artificial neural network (ANN) method and machine learning.^23^ Structural analysis identifies neoepitopes by calculating the minimal free energy of epitope‐HLA complex.^24^, ^25^ PSSM is produced by measuring the interaction between peptides and specific MHC molecule.^26^ The correlation of different positions in sequence was considered into ANN analysis to predict affinity between peptides and MHC molecule. Machine learning could predict affinity of peptides and MHC molecule by learning the affinity of known functional regions with peptides. The immune epitope database (IEDB) predicts the optimal amino binding positions of MHC‐I molecule through a large variety of HLA allele algorithms, thereby being broadly applicated for identifying the epitope peptides.^27^ Additionally, HLA ligandome approach could identify naturally HLA‐presented peptides existed in tumour cells by mass spectrometry analysis.^28^ It could also be used to identify specifically overexpressed protein‐derived peptides, signal peptide‐derived peptides and antigenic mutation‐derived peptides.^29^ This approach could combine with computational biology and bioinformatics, such as functional annotation and gene expression analysis, to identify potential TSA (including neoantigens) and TAA. Based on ligandome analysis, we can observe a few peptides of 11 amino acids, 12 amino acids and 13 amino acids, as their length is outside the consensus of the computer programs for motif prediction of class I peptides.

**TABLE 2:** In silico analysis for peptide‐based therapeutic cancer vaccines

Year	Database	Method	Methodology	Website	Characteristics
1994	BIMAS	Experimental verification	A total of 154 peptides were combined together to generate a table containing 180 coefficients (20 amino acids x 9 positions), each of which represents the contribution of one particular amino acid residue at a specified position within the peptide to binding to HLA‐A2. Provides a predicted t1/2 of dissociation	http://www‐bimas.cit.nih.gov / molbio / hla_bind	
1997	SYFPEITHI	Experimental verification	The algorithm takes into account a number of characteristics of both the HLA haplotype as well as the peptide of interest, and uses these data to provide a binding score	http:// /www.syfpeithi.de /	
2000	IEDB	Experimental verification	The IEDB combined all published data associated with epitopes and a large scale of experimentally determined peptides	www.immuneepitope.org	IEDB could provide consistent and accurate data with improved interoperability
2002	RANKPEP	PSSM	The binding potential of any peptide sequence (query) to a given MHC molecule is linked to its similarity to a group of aligned peptides known to bind to that MHC	www.mifoundation.org/Tools/ rankpep.html	
2005	NetCTL	Bioinformatics prediction	Integration the predictions of proteasomal cleavage, TAP transport efficiency and MHC class I affinity	http://www.cbs.dtu.dk/services/NetCTL	
2008	NetMHC	PSSM	The software integrates affinity measurements of IEDB database and data of eluting ligands in SYFPEITHI database to train 55 MHC allele‐specific artificial neural networks and additional position‐specific scoring matrix (PSSM) of 67 HLA allele	http://www.cbs.dtu.dk/services/NetMHC.	The binding affinity measurements of 8‐, 10‐ and 11‐mer were predicted based on properties of 9‐mer
2009	PMBEC	PSSM	PMBEC is derived from the binding affinity data of combinatorial peptide mixtures to build up matrix properties of amino sequence		The software could compensate for missing information on specific residues in the training data
2015	ANN‐Hydr	Machine learning			Training on a relative hydrophobicity scale

Abbreviations: ANN, artificial neural network; BIMAS, bioinformatics and molecular analysis section; CTL, cytotoxic T lymphocyte; HLA, human leucocyte antigen; IEDB, the immune epitope database; MHC, major histocompatibility complex; PSSM, position‐specific scoring matrix; TAP, transporter associated with antigen processing.

## 4 CLINICAL APPLICATION OF PEPTIDE‐BASED THERAPEUTIC CANCER VACCINES

Since Hu et al reported that MAGE‐1 (melanoma antigen‐1)‐derived peptide can be used as peptide‐based therapeutic cancer vaccine in clinical trial, various TA‐derived epitopes have been identified for clinical application of peptide‐based vaccines.^30^ Most recently, peptide‐based vaccines are tested in clinical trials for multiple cancers, including melanoma,^31^ oesophageal cancer,^32^, ^33^ lung cancer,^34^, ^35^ pancreatic cancer,^36^ and head and neck squamous cell carcinoma.^37^ The study of Mittendorf et al^38^ showed that E75 (nelipepimut‐S), a HLA‐A2/A3‐restricted immunogenic peptide‐derived HER2, was safe and appeared to have clinical efficacy. And a phase III has been initiated. Mittendorf et al also reported the phase II trial evaluating GP2 (a HER2‐derived, HLA‐A2^+^ restricted peptide) + GM‐CSF (granulocyte‐macrophage colony‐stimulating factor) setting to breast cancer patients to prevent recurrence. Results suggested that the vaccine might be effective in patients with HER2‐positive tumours who also received trastuzumab.^39^ Phase II trial of a multivalent WT1 peptide vaccine (galinpepimut‐S) in leukaemia^40^ and phase I/II trial of MUC1, HER2 and CEA (carcinoembryonic antigen) HLA‐A2^+^‐restricted peptides^41^ also showed that peptide‐based vaccines were feasible, safe and well tolerated. Sipuleucel‐T for prostate cancer was the first peptide‐based therapeutic cancer vaccine approved to go to the market by Food and Drug Administration (FDA).

The peptide‐based therapeutic cancer vaccines in clinical trials often combine multiple targets with multiple epitopes by different screening strategies, unlike in vitro studies that usually focus on a single antigen. Due to the presence of multiple epitopes, T cells that recognize different targets can be activated to minimize tumour immune escape caused by antigen loss. Moreover, the combination of HLA‐I and HLA‐II class epitopes increases the possibility of both CD4^+^ and CD8^+^ effector T‐cell activation, which contributes to the persistence and survival of effector cells in vivo.^42^ Therefore, these peptide‐based therapeutic cancer vaccines have been reported to be well tolerated and have shown clinical benefits against tumours. In the following paragraphs, we focused on introductions of targets, sequences and research progress of epitope peptides in recent 5 years (Table 3).

**TABLE 3:** Clinical trials of peptide‐based therapeutic cancer vaccines in recent five years

Tumour	Targets	Epitopes	Reference	Phase
Solid tumour	GPC3	FVGEFFTDV	^92^	Ⅰ
	KOC1, DEPDC1, MPHOSPH1, TTK, URLC10	KTVNELQNL, EYYELFVNI, IYNEYIYDL, SYRNEIAYL, RYCNLEGPPI	^48^	Ⅰ
	WT1	CYTWNQMNL	^66^	I/II
Melanoma	Tyrosinase, gp100, MART‐1	YMDGTMSQV, IMDQVPFSV, LAGIGILTV	^93^	Ⅲ
Breast cancer	HER2	IISAVVGIL	^39^	I/II
		E75(nelipepimut‐S, KIFGSLAFL)	^38^	I/II
		IISAVVGIL, LRMKGVGSPYVSRLLGICL	^94^	II
Breast cancer, ovarian cancer	MUC1, ErbB2, CEA	SAPDNRPAL, KIFGSLAFL, YLSGADLNL	^41^	I/II
Leukaemia	WT1	YMFPNAPYL, RSDELVRHHNMHQRNMTKL, PGCNKRYFKLSHLQMHSRKHTG, SGQAYMFPNAPYLPSCLES	^40^	I/II
		KRYFKLSHLQMHSRKH		Ⅰ
Renal carcinoma	APOL‐1, APOL‐2, KIAA0367, ITGA3, MUC‐1, ADFP, MET, CCND1, RGS5, GUCY1A3	FLGENISNFL, ALADGVQKV, ALFDGDPHL, SVFAGVVGV, LLYPTEITV, STAPPVHNV, SVASTITGV, YVDPVITSI, LAALPHSCL, LLGATCMFV	^95^	I/II
	HIG2	VLNLYLLGV	^96^	Ⅰ
Glioma	ANKRD40, BCA, CDK4, EIF4E, PTP, USP11, et al	33 HLA‐A*02:01‐binding and 26 HLA‐A*24:02‐binding peptides	^63^	Ⅰ
	WT1	CYTWNQMNL, KRYFKLSHLQMHSRKH	^56^	I/II
	Survivin	DLAQMFFCFKEL	^97^	Ⅰ
	BCAN, CHI3L2, CSPG4, FABP7, IGF2BP3, NLGN4X, NRCAM, PTPRZ1, TNC	ALWAWPSEL, SLWAGVVVL, TMLARLASA, LTFGDVVAV, KIQEILTQV, NLDTLMTYV, GLWHHQTEV, AIIDGVESV, KVFAGIPTV, AMTQLLAGV		
Neuroblastoma	NY‐ESO‐1	SLLMVVITQV	^49^	
Colorectal cancer	RNF43, TOMM34	NSQPVWLCL, KLRQEVKQNL	^98^	II
	RNF43, TOMM34, KOC1, VEGFR1/2	NSQPVWLCL, KLRQEVKQNL, KTVNELQNL, SYGVLLWEI, RFVPDGNRI	^99^	II
Oesophageal cancer	DEPDC1, MPHOSPH1, URLC10, CDCA1, KOC1	EYYELFVNI, IYNEYIYDL, RYCNLEGPPI, KTVNELQNL, YMMPVNSEV, KLATAQFKI	^43^	
Colorectal cancer, oesophageal cancer	HSP105	NYGIYKQDL, EYVYEFRDKL, RLMNDMTAV, KLMSSNSTDL	^100^	Ⅰ
Gastric cancer	FOXM1, DEPDC1, KIF20A, URLC10, VEGFR	IYTWIEDHF, RYCNLEGPPI, EYYELFVNI, KVYLRVRPLL, SYGVLLWEIF	53, 55	I/II
Gastrointestinal cancer	HSP70, GPC3	YGAAVQAAI, MVNELFDSL	^91^	Ⅰ
Pancreatic cancer	KIF20A, VEGFR1/2	KVYLRVRPLL, SYGVLLWEI, RFVPDGNRI	^90^	II
	WT1	RMFPNAPYL, CYTWNQMNL	^61^	II
Lung cancer	IDO	ALLEIASCL	^47^	Ⅰ
Bladder cancer	DEPDC1, MPHOSPH1	EYYELFVNI, MVNELFDSL / LFDSLFPVI / SLQVTRIFL	^44^	I/II
Prostatic cancer	Personalized peptide vaccination (PPV)	LLQAEAPRL / KLKHYGPGWV / KLVERLGAA / DVWSFGILL / DLLSHAFFA / ASLDSDPWV / RLQEWCSVI / NVLHFFNAPL / DYSARWNEI/VYDYNCHVDL/HYTNASDGL/DYLRSVLEDF/RYLTQETNKV/LYCESVHNF/HYRKWIKDTI/DYVREHKDNI/WLEYYNLER/QIRPIFSNR/ILEQSGWWK/VIQNLERGYR/GIHKQKEKSR/GAAPLILSR/APAGRPSASR/KIREEYPDR	^83^	II
	CDCA1	VYGIRLEHF	^62^	Ⅰ
Cervical carcinoma	FOXM1, MELK, HJURP, VEGFR1/2	YLVPIQFPV, SLVLQPSVKV, GLMDLSTTPL, RFVPDGNRI	^57^	Ⅰ
Ovarian cancer	FBP	EIWTHSYKV / EIWTFSTKV	^64^	I/II

### 4.1 Study design and treatment

Peptide‐based therapeutic cancer vaccines are usually administered in a 7‐ to 15‐day interval with subcutaneous axillary and/or inguinal injection of 1‐3 mg/dose per peptide per person. Patients usually complete a course of at least 2 months to a maximum of 12 months unless patients experience disease progression or unacceptable toxicity. The primary end points are safety, tolerability, immunogenicity and operational feasibility of the peptide‐based vaccines. The secondary end points are evaluations of anti‐tumour effects, overall survivals (OS) and disease‐free survivals (RFS).

### 4.2 Clinical efficacy and immune response

Analysis on patients treated with peptide‐based vaccines showed that the production of epitope‐specific CTLs could be induced in most patients, and even tumour infiltrating lymphocyte (TIL) activation could be induced in individual patients.^43^ The CD8^+^ T cells in lymph nodes and the infiltration of CD8^+^ T cells in the tumour microenvironment increased in about 30%‐60% of patients, and the secretion of granzyme B and interferon‐γ (IFN‐γ) also increased. Patients who showed a strong epitope‐specific CTL response had longer OS than those with non‐ or low immune response, demonstrating that peptide‐based vaccines could be effective in patients who showed a peptide‐specific immune response. Compared with the placebo group, patients receiving the peptide‐based vaccine showed a tendency of improved OS and RFS, and their condition was more stable. The peptide‐based vaccine therapy usually shows delayed immune response and tumour growth inhibition, but does not show significant tumour shrinkage.^44^, ^45^ Additionally, the epitope peptide could induce anti‐tumour response over a long period of time.^46^ Kjeldsen et al reported that 13.3% of patients showed anamnestic immune response 6 years after primary immunization.^47^ In another case of oesophageal cancer, the patient received 8 vaccinations every 6 months, a total of 38 vaccinations, and finally obtained a complete response (CR) lasting for 5 years.^48^ Although peptide‐specific responses also were elicited in high‐risk patients, previous studies showed that patients in the early stage of tumour progression or with a low disease burden could obtain better clinical benefits.^49^, ^50^, ^51^ This is because the immunosuppressive tumour microenvironment was the stronger in high‐risk patients compared with low‐risk patients. There were no significant differences in OS and RFS between the vaccine treatment group and the control group in some clinical trials. For example, Brian IRini et al reported that the peptide‐based vaccine did not improve any clinically relevant indicators in advanced metastatic renal cancer in a phase III study.^52^ Possible reasons for lack of clinical benefits include the patient's low immune status, the limited response to the vaccine, and the poor dose and/or the short duration of treatment. The clinical effect of peptide‐based vaccine may be delayed compared with chemotherapy due to the mechanism of immune response, which may lead to a longer observation period to evaluate the clinical benefits. Some studies also recruited patients with advanced disease who were resistant to multiple chemotherapies, and it was difficult for these patients to gain clinical benefits from the peptide‐based vaccines due to the poor state of their immune systems. Therefore, peptide‐based vaccines may be suitable as an adjuvant therapy for cancer patients after surgery.^53^

### 4.3 Adverse events

The peptide‐based vaccines have distinct characteristics of better tolerance and safety compared with conventional anti‐tumour therapies, such as chemotherapy and immune checkpoint inhibitors, and the vaccines generally could not cause serious systemic adverse events (AEs). The most common AEs related to the peptide‐based vaccine are erythema and induration related to the injection site with grade 1 or 2,^53^ which are easy to be reversed. Patients with reaction at the injection sites (RAI) generally showed a better prognosis than those without skin reaction,^53^, ^54^ suggesting that RAI might be a surrogate predictor of CTL response to peptide‐based vaccine. Other grade 1 or 2 AEs include nausea, diarrhoea, myalgia, fatigue, increased aspartate aminotransaminase, and increased blood alkaline phosphatase^55^, ^56^, ^57^ and urinary irritation in bladder cancer.^58^ No dose‐related toxicity and treatment‐related death were observed. Some studies reported grade 1‐3 AEs in haematology, such as hypoalbuminemia, thrombocytopenia, leukopenia, neutropenia, anaemia and bone marrow suppression, which were mainly related to the cancer progression.^57^, ^59^, ^60^, ^61^, ^62^, ^63^ However, the causal relationship between anaemia and the peptide‐based vaccine cannot be ruled out in the vaccine targeting VEGFR.^55^ Moreover, the most common grade 3 or higher AEs were RAIs (including ulcers and diffuse maculopapular rash) and headache. The peptide‐based vaccine‐related grade 3 AEs included chest pain, dyspnoea and pulmonary embolism, which may be due to the expression of epitope‐related TA in lung tissue, leading to a direct immune response (on‐target and off‐tumour).^64^, ^65^ During the six‐year follow‐up, IDO (indoleamine 2,3‐dioxygenase)‐specific peptide vaccine showed no grade 3 or 4 AEs, which ensured the long‐term safety of peptide‐based vaccines.^47^ Sawada et al found the TA‐specific CD8^+^ T cells showed exhausted phenotypes in individual patients, which may be due to over‐activation of CD8^+^ T cells in patients with high tumour mutation burden or over‐frequent vaccinations.^66^ In summary, patients could gain clinical benefits from peptide‐based therapeutic cancer vaccines with distinct advantages of safety, good tolerance and effective immunization.

## 5 COMMON PHARMACEUTICAL FORMULATIONS OF PEPTIDE‐BASED THERAPEUTIC CANCER VACCINES

The peptide‐based therapeutic cancer vaccines can improve the prognosis of cancer patients, while a more effective vaccine is needed to improve PFS and OS of patients. One of the strategies is developing a safe and effective immune formulation to enhance TA‐derived peptide‐specific immunity. The epitope peptides with instinct features of low molecular weight, easy to degradation and short half‐life accelerated the development of pharmaceutical formulations of peptide‐based vaccines. The preparation of formulations usually by prolongating epitope persistence, enhancing co‐stimulation signal, increasing local inflammation and triggering non‐specific proliferation of lymphocytes enhanced the efficacy of peptide‐based vaccines. These formulations can be divided into immune stimulation adjuvants and vaccine delivery systems according to the main mechanism of action.

### 5.1 Immune stimulation adjuvants

Immune stimulation adjuvants could enhance humoral immune and Ⅳ type allergy to induct IFN‐γ secretion, regulate MHC‐II class antigen expression for producing TA‐specific CTLs, such as complete Freund's adjuvant, incomplete Freund's adjuvant (IFAs), toll‐like receptor (TLR) agonists and cytokines. The incomplete Freund's adjuvant, Montanide ISA (incomplete Seppic adjuvant) 51, and the cytokine, GM‐CSF, are widely used in clinical trials.

#### 5.1.1 Montanide ISA 51

**FIGURE 3:**
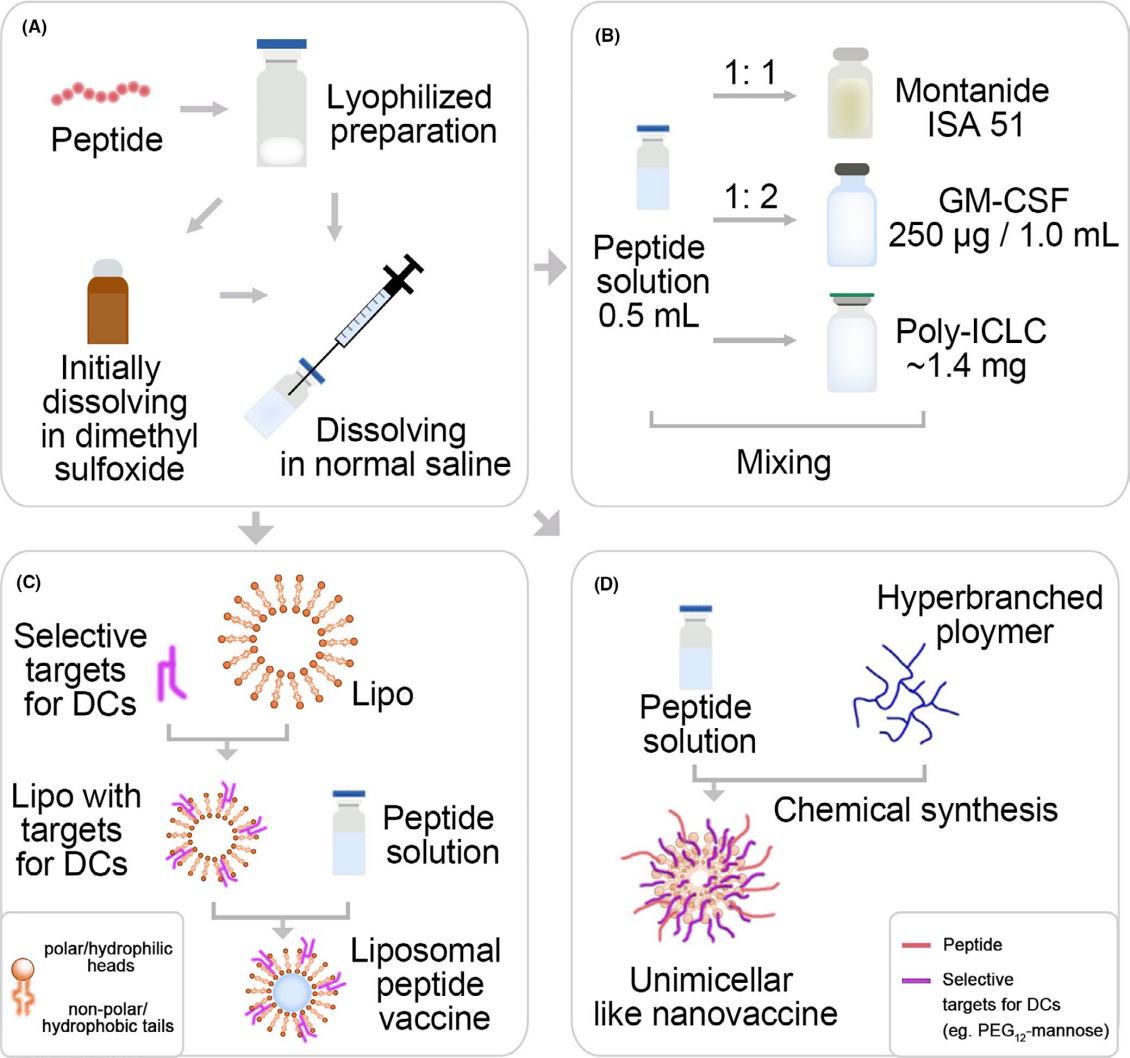
Preparing emulsions, micelles and nanoparticles for epitope peptides. a, Dissolution of lyophilized preparation of epitope peptides. **b,** Epitope peptides mixing with immune adjuvants. **c,** Design diagram of liposomal peptide vaccine. **d,** Peptide amphiphile micelles for vaccine delivery

Not only could Montanide ISA 51 trigger immune responses, but also enhance the depot effect of vaccines. Due to the non‐absorbable mineral oil composition, it remains at the subcutaneous injection site for weeks to months, helping maintain persistence of epitopes to active T cells.^67^ Combining the epitope peptides with Montanide ISA 51 may cause a stronger immune response and kill more tumour cells. Before vaccination, lyophilized powder of epitope peptides was dissolved in the appropriate solvent, such as normal saline or dimethyl sulphoxide diluted with normal saline (Figure 3A). Then, solvent mixed with Montanide ™ ISA 51 VG (Seppic Inc, Paris, France) at ratio of 1:1 until the two liquids generating a thick, creamy, opaque and consistent emulsion (Figure 3B). The patients are usually vaccinated at a 1.0 mL dose level containing 1‐3 mg epitopes. Valmori D et al tested different formulations to improve the CTL immune response. The results showed that IFA injection significantly increased the CTL response.^36^ Sher YP et al used Montanide ISA 51 combined with Th epitopes derived from TAL6 antigen and CpG ODN (cytosine guanine oligodeoxynucleotide, TLR9 agonist) to make the immune stimulation adjuvant, showing that the epitope with adjuvant was more effective in inhibiting tumour growth and metastasis than the epitope alone.^68^ Although Montanide ISA 51 is generally safe, it often causes local side effects, such as skin irritation and inflammation, even ulcers.

#### 5.1.2 GM‐CSF

GM‐CSF is usually utilized as an adjuvant due to it can enhance effective priming of T‐cell responses by attracting and stimulating DCs in skin loaded with tumour‐associated epitope peptides upon vaccination. It may also have antiangiogenic activity and has been successfully applied in late‐stage clinical trials. The lyophilized powder of peptides was dissolved in 0.5 mL suitable solvent and mixed with 250 μg / 1.0 mL GM‐CSF, and the total volume was 1.5 mL (Figure 3B).^38^ Previous studies of peptide‐GM‐CSF clinical trials demonstrated that side effects could be attributed to the toxicity of GM‐CSF rather than the immune activity of peptides,^39^, ^69^ and the most serious side effect was only RAI.

#### 5.1.3 TLR agonist

TLR agonists are effective adjuvants that could enhance epitopes‐induced CTL memory activation.^70^ TLR3 agonist poly‐ICLC (lysine and carboxymethylcellulose) is widely used to stimulate tumour‐specific T‐cell response to prevent T cell from exhausting and to improve immunotherapy outcomes.^71^ The vaccines with combination of epitope solutions and 1.4 mg poly‐ICLC could effectively induce epitope‐specific CTL activity (Figure 3B). Melssen et al^72^ reported poly‐ICLC can be used as an effective vaccine adjuvant to induce CD8^+^ T‐cell immune response with targeting action and acceptable safety. TLR4 agonists as vaccine adjuvants have also been used in clinical trials, but the classic TLR4 agonist LPS (lipopolysaccharides) has been considered to be toxic. Besides, CD8^+^ T‐cell immune response induced by poly‐ICLC may be marginally more responsive than LPS.

### 5.2 Vaccine design and delivery system

Optimized delivery systems have been developed to design rational vaccines, which usually consist of comparable size, such as liposomes, microemulsions, immune‐stimulating complexes, and other nanometre or microparticle systems. The delivery system being especially suitable for the development of vaccines could improve clinical benefits of vaccines.

In recent years, more and more attention has been paid to the design of peptide‐based nanoparticle vaccines for tumour immunotherapy (Figure 3C). The optimized liposome‐based vaccines could co‐deliver peptides and adjuvants to promote their delivery to lymphoid organs and to draining lymph nodes (dLNs), which shows the acceptable clinical potential of liposome as delivery system.^73^ The bioconjugation strategy links the target to the particle to improve the peptides/adjuvant co‐delivery to the DCs in lymph nodes for immune response enhancement. Additionally, liposomes can encapsulate multiple epitopes to target different TAs, which can better meet the needs of clinical application. Rueda F et al^74^ used liposomes to encapsulate B epitopes, T‐cell epitopes, Th epitopes and TLR ligands to improve the immunity of the vaccine. Arab A et al^75^ developed effective vaccine delivery/auxiliary systems by connecting the epitope E75, which was derived from the highly expressed antigen HER2 in breast cancer patients, with the liposome containing distearoyl phosphatidylcholine (DSPC) and distearoyl phosphatidylglycerole (DSPG). Martine A et al^68^ also developed liposome‐based co‐delivery system containing melanoma‐associated antigen‐derived peptide GP100280‐288 and TLR4 ligand monophosphoryl lipid A (MPLA), which could be phagocytized by subcutaneous DCs and significantly enhanced the epitope‐specific T‐cell response. These results indicated that strategy of nanocarriers based on liposome is effective to induce anti‐tumour immune response.

Similarly, unimicellar nanostructures based on amphiphilic dendrimers, hyperbranched polymers and cross‐linked block copolymer micelles are another acceptable strategy, which could not depolymerize when diluted. Additionally, Rui Zhang et al^76^ reported that the antimicrobial peptide with low toxic cholesterol modification, DP7‐C, showed a dual role as carrier and immune adjuvant. DP7‐C with hydrophilic DP7 and hydrophobic cholesterol could self‐assemble into amphiphilic micellar structure in aqueous solution, improving the efficacy of DC‐based vaccines (Figure 3D). The toxicity of peptide‐based vaccines may be related to the membrane instability caused by the hydrophobicity of peptides, which can be reduced by fusing the peptides with the polymer into the micellar structure.^72^ In general, the micelle‐based could elicit the significant immune response to inhibit tumour growth.

Short peptide‐based supramolecular hydrogel with three dimensional networks of nanofibres, nanotubes and nanoparticles^77^ was a novel and promising immunostimulant, which could improve the biostability and bioactivity of peptides. The hydrogel formulation could protect the peptide against enzyme digestion and nanofibres in gels facilitated the uptake of peptides by DCs, thereby increasing the accumulation of peptides in lymph nodes to activate immune response. Yang et al^78^ describe a supramolecular hydrogel of a self‐assembling D‐tetra‐peptide capable of evoking both humoral and cellular immune responses. The D‐tetra‐peptide (Nap‐GFFY) could form hydrogels by a heating‐cooling process or simply by an autoclave in phosphate‐buffered saline (PBS, pH 7.4), and allow the incorporation of different peptides by mixing through vortex or shaking. Moreover, the Nap‐GDFDFDYTKPR hydrogel discovered on this basis combined tuftsin (TKPR) and Nap‐GDFDFDY, which showed an excellent anti‐tumour efficacy by stimulating a powerful CD8^+^ T‐cell immune response, enhancing the phagocytic activity of macrophages and promoting the maturation of DCs.^79^ Due to the very simple preparation process, the good biocompatibility and strong vaccine adjuvant potency, short peptide‐based supramolecular hydrogel suggested a great potential in vaccine development.

## 6 COMBINATION OF PEPTIDE‐BASED THERAPEUTIC CANCER VACCINES AND OTHER THERAPIES

Although many studies have demonstrated the effectiveness of peptide‐based therapeutic cancer vaccines, no vaccine has shown significant OS benefits in randomized phase III clinical trials. However, combination of therapies aimed at controlling immune tolerance might improve outcomes, such as chemotherapy, radiotherapy (RT), biological agents and immune checkpoint inhibitors (Table 4). In addition to TA‐derived peptide vaccination, the personalized peptide vaccination (PPV), a novel immunotherapeutic approach based on a specific pool of peptides, is usually used on the combination strategy with other therapies in clinical trials. The peptide pool of PPV includes all information on the HLA‐A type, and the peptide candidate library includes mutated peptides and highly expressed peptides. Considering the heterogeneous antigen expressions of different patients before vaccination, four specific epitopes aiming to the individual patient were selected from the candidate peptides into combination application strategy of peptide‐based therapeutic cancer vaccines.

**TABLE 4:** Therapeutic value of combined application of peptide‐based vaccines and other therapies in recent five years

Combined classification	Combined drug	Tumour	Targets	Epitopes	Combined effect	Reference	Phase
Chemotherapy	Cyclophosphamide	Solid tumour	RNF43	ALWPWLLMAT / ALWPWLLMAT	Decreased ratio of Tregs, increased tumour‐specific immune responses and clinical efficacy	^81^	Ⅰ
			KOC1, DEPDC1, MPHOSPH1, TTK, URLC10	KTVNELQNL, EYYELFVNI, IYNEYIYDL, SYRNEIAYL, RYCNLEGPPI	Decreased ratio of Tregs	^48^	Ⅰ
		Cholangiocarcinoma	CypB, NRPL, p56Lck, ppMAPkkk, SART3, UBE2V, WHSC2	KLKHYGPGWV; ALVEFEDVL; NVLHFFNAPL; KLVERLGAA; DVWSFGILL; DLLSHAFFA; LLQAEAPRL; RLAEYQAYI; RLQEWCSVI; LIADFLSGL; ASLDSDPWV; ILGELREKV	T‐cell response enhancement, significant PFS and OS elongation	^82^	II
	Docetaxel	Lung Cancer	PPV	12 peptides for HLA‐A2, 14 peptides for HLA‐A24, 9 peptides for HLA‐A3, and 4 peptides for HLA‐A26	No survival improvement	^101^	II
	Gemcitabine	Pancreatic cancer	WT1	RMFPNAPYL / CYTWNQMNL	PFS and OS elongation	^61^	II
			KIF20A, VEGFR1/2	KVYLRVRPLL, SYGVLLWEI, RFVPDGNRI	Good tolerance, clinical benefits	54, 102	II
	5‐Fluorouracil, cyclophosphamide, levofolinic acid, oxaliplatin	Colorectal cancer	TS	YMIAHITGLFLDSLGFSTTLGDAHIYL	Lymphocytes response enhancement, PFS and OS elongation	^103^	Ⅰ
	Dexamethasone	Prostate cancer	PPV: SART3, Cyclophilin B, p56lck, ppMAPkkk, WHSC2, UBE2V, HNRPL, SART2, MRP3, PAP, PSA, EGF‐R, IEX‐1, β‐tublin5	LLQAEAPRL / KLKHYGPGWV / KLVERLGAA / DVWSFGILL / DLLSHAFFA / ASLDSDPWV / RLQEWCSVI / NVLHFFNAPL / DYSARWNEI / VYDYNCHVDL / HYTNASDGL / DYLRSVLEDF / RYLTQETNKV / LYCESVHNF / HYRKWIKDTI / DYVREHKDNI / WLEYYNLER / QIRPIFSNR / ILEQSGWWK / VIQNLERGYR / GIHKQKEKSR / GAAPLILSR / APAGRPSASR / KIREEYPDR	Significant OS elongation	^98^	II
	Platinum‐containing chemotherapy plus best supportive care	Bladder cancer	PPV	31 candidate peptides for patients with positive HLA‐A2, ‐A3, ‐A11, ‐A24, ‐A26, ‐A31 or ‐A33 alleles	OS elongation	^84^	II
Radiotherapy	Radiotherapy	Liver cancer	PPV	P1: CORE‐18, MUC‐12, KRAS‐A02‐G13D1, PSCA‐76 P2: PI3KCA‐A02‐H1047L‐1, CORE‐35, WTP53‐149, AFP‐137 P3: EGFR‐800, KRAS‐A11‐G13D, CYPB‐84, CTNNB1‐A11‐S45F P4: KRAS11‐12C, EGFR‐54,AFP‐403, Survivin28‐80 P5: AFP‐357, VEGFR2‐169, KRAS‐A11‐12C, MRP3‐1293 P6:KRAS‐A11‐12D, CTNNB1‐A11‐41A, CTNNB1‐A11‐S45F, KRAS‐A11‐12R P7:SART3‐109, CORE‐18, PSCA‐7, hTERT‐540 P8:AFP‐357, KRAS‐A11‐12D, VEGFR2‐169, PSCA‐776 P9:CTNNB1‐A11‐S45F, CTNNB11‐41A, CTNNB11‐45P, EGFR‐54	Regression of tumour, decrease of AFP level	^85^	Ⅰ
Targeted therapy	Trastuzumab	Breast cancer	HER_2_	E75(nelipepimut‐S, KIFGSLAFL)	No added cardiac toxicity	^88^	II
Biological agents	Bacillus Calmette‐Guérin	Bladder cancer	DEPDC1, MPHOSPH1	EYYELFVNI, IYNEYIYDL	Good tolerance	^58^	II

### 6.1 The effect of combined chemotherapy and peptide‐based vaccine

Causes of low immune responses may be associated with high Treg number. Since cyclophosphamide could selectively deplete Tregs^80^ and regulate dendritic cell homoeostasis, the combination of low‐dose cyclophosphamide and peptide‐based therapeutic cancer vaccines may provide clinical benefits.^81^, ^82^ However, the peptide‐based vaccines combined with low‐dose IL‐2 (interleukin‐2) may exert negative effects on anti‐cancer therapies due IL‐2 may increase Tregs.^48^ In addition, compared with Treg inhibitor gemcitabine alone, more than half of patients treated with peptide‐based vaccine combined with gemcitabine showed long‐lasting epitope‐specific T‐cell immune responses, reduced tumour burden, and long‐term stable disease.^61^ However, the peptide‐based vaccine in combination with gemcitabine was not effective in patients with advanced metastatic disease, which was consistent with the opinion that the optimal condition for obtaining long‐term clinical benefits was in the early stage of tumour or with a low disease burden described above. Besides, for prostate cancer patients treated with peptide‐based vaccine and low‐dose dexamethasone, OS was significantly prolonged compared with dexamethasone alone due to induction of the specific anti‐tumour immunity.^83^ In addition, OS also appeared to be improved when combined with peptide‐based vaccines and platinum drugs.^84^

### 6.2 The effect of combined radiotherapy and peptide‐based vaccine

The radiation may not reach all tumour focuses due to metastases or the large size of the tumour during radiotherapy. The combination of radiotherapy and peptide‐based vaccines can effectively prevent tumours.^85^ Release of danger‐associated molecular patterns by RT‐induced cell death, resulting in the facilitation of tumour antigen uptake by DCs and cross‐presentation on MHC class I, is the molecular mechanism by which the combination strategy modifies the tumour microenvironment and enhances anti‐tumour immune response. The other advantage is that the combination strategy is expected to reduce the dosage of chemotherapy drugs to avoid the side effects of chemotherapy, which has great potential clinical application values.

### 6.3 The effect of combined other antineoplastic agents and peptide‐based vaccine

The combination of anti‐HER2 antibody trastuzumab with the HER2‐targeting peptide‐based vaccine in preclinical studies led to the proliferation of peptide‐specific CTLs due to trastuzumab‐induced improvement of cross‐presentation of HER2 epitope‐pulsed DCs.^86^, ^87^ Clifton et al proved that the combination of HER2‐targeting peptide vaccine nelipepimut‐S and trastuzumab is well tolerated. Cardiac dysfunction of class III or IV was observed in the phase III trial of trastuzumab, and the combination of trastuzumab and HER2‐derived peptide vaccine did not increase the cardiotoxicity.^88^

Upregulation of immune checkpoint molecule expression on CD8^+^ T cells, such as PD‐1 (programmed death 1), TIM‐3 (T‐cell immunoglobulin mucin 3) and TIGIT (T‐cell immunoreceptor with Ig and ITIM domains), could inhibit immunopotentiation of the peptide‐based vaccine. The peptide‐based vaccine could also promote the infiltration of CD45RO^+^ activation/memory T cells into the tumours, which in turn facilitate the increase of PD‐1^+^ TILs.^89^ These suggested that combination strategy of immune checkpoint inhibitors and peptide‐based vaccines may be beneficial for tumour patients.^90^, ^91^ Indeed, the emergent of preclinical and clinical data demonstrated that the anti‐tumour activity of immune checkpoint inhibitors can be enhanced by peptide vaccination.

## 7 CONCLUSION AND PERSPECTIVE

The peptide‐based therapeutic cancer vaccines could be well equipped with easy manufacturing, excellent safety profiles and low cost compared with lentivirus‐transduced DC vaccine. The FDA‐approved HLA‐restricted epitope also demonstrated that the strategy based on immune response could revolutionize cancer treatments. Unfortunately, many studies about peptide‐based vaccines have failed in clinical trials due to the immunoevasion of tumour cells and the loss of tumour antigen. Some ‘CTL epitopes’ with low immunogenicity cannot be effectively cross‐presented by DCs in vivo to favour cross‐priming of CTLs. Therefore, it is important to further identify and optimize epitopes with immunogenicity for clinical application.

Despite the peptide‐based cancer vaccines with specific cytotoxicity against tumour cells, there are major challenges of inducing continuous and high immune response level. The results of the early clinical trials thus far conducted suggested that the peptide‐specific immunity gradually decreased over time. The FDA guidelines point that multi‐target vaccines targeting different tumour antigens could generate multiple TA‐specific immune responses, which are expected to overcome resistance of peptide‐based vaccines to effectively inhibit tumour immunoevasion. Therefore, the novel strategy emerging on the identification of epitopes derived from TAs associated with tumour progression can contribute to the development of multi‐target vaccines and improve the efficacy of peptide‐based vaccines. We hope that multi‐peptides therapeutic cancer vaccines could offer a powerful potential in future clinical application at the era of successful immunotherapy.

## CONFLICTS OF INTEREST

The author declares that he/she has no competing interests.

## Data Availability

All data generated or analysed during this study are included in this published article.
